# *Ace2* and *Tmprss2* Expressions Are Regulated by *Dhx32* and Influence the Gastrointestinal Symptoms Caused by SARS-CoV-2

**DOI:** 10.3390/jpm11111212

**Published:** 2021-11-16

**Authors:** Fuyi Xu, Jun Gao, Buyan-Ochir Orgil, Akhilesh Kumar Bajpai, Qingqing Gu, Enkhsaikhan Purevjav, Athena S. Davenport, Kui Li, Jeffrey A. Towbin, Dennis D. Black, Joseph F. Pierre, Lu Lu

**Affiliations:** 1 School of Pharmacy, Binzhou Medical University, Yantai 264003, China; xufuyiphd@gmail.com; 2 Department of Genetics, Genomics and Informatics, University of Tennessee Health Science Center, Memphis, TN 38163, USA; gjsaas@gmail.com (J.G.); akhil.bajpai@gmail.com (A.K.B.); qgu4@uthsc.edu (Q.G.); astarlar@uthsc.edu (A.S.D.); 3 Institute of Animal Husbandry and Veterinary Science, Shanghai Academy of Agricultural Sciences, Shanghai 201106, China; 4 Department of Pediatrics, University of Tennessee Health Science Center, Memphis, TN 38163, USA; borgil@uthsc.edu (B.-O.O.); epurevja@uthsc.edu (E.P.); jtowbin1@uthsc.edu (J.A.T.); dblack@uthsc.edu (D.D.B.); 5 Children’s Foundation Research Institute, Le Bonheur Children’s Hospital Memphis, Memphis, TN 38103, USA; 6 Department of Microbiology, Immunology, and Biochemistry, University of Tennessee Health Science Center, Memphis, TN 38163, USA; kli1@uthsc.edu; 7 Pediatric Cardiology, St. Jude Children’s Research Hospital, Memphis, TN 38105, USA

**Keywords:** BXD mice, gastrointestinal tract, transcriptome, co-expression, COVID-19, SARS-CoV-2, systems genetics, microbiota

## Abstract

Studies showed that the gastrointestinal (GI) tract is one of the most important pathways for SARS-CoV-2 infection and coronavirus disease 2019 (COVID-19). As SARS-CoV-2 cellular entry depends on the ACE2 receptor and TMPRSS2 priming of the spike protein, it is important to understand the molecular mechanisms through which these two proteins and their cognate transcripts interact and influence the pathogenesis of COVID-19. In this study, we quantified the expression, associations, genetic modulators, and molecular pathways for *Tmprss2* and *Ace2* mRNA expressions in GI tissues using a systems genetics approach and the expanded family of highly diverse BXD mouse strains. The results showed that both *Tmprss2* and *Ace2* are highly expressed in GI tissues with significant covariation. We identified a significant expression quantitative trait locus on chromosome 7 that controls the expression of both *Tmprss2* and *Ace2*. *Dhx32* was found to be the strongest candidate in this interval. Co-expression network analysis demonstrated that both *Tmprss2* and *Ace2* were located at the same module that is significantly associated with other GI-related traits. Protein–protein interaction analysis indicated that hub genes in this module are linked to circadian rhythms. Collectively, our data suggested that genes with circadian rhythms of expression may have an impact on COVID-19 disease, with implications related to the timing and treatment of COVID-19.

## 1. Introduction

The ongoing COVID-19 pandemic, caused by severe acute respiratory syndrome coronavirus 2 (SARS-CoV-2), poses a major global health challenge [1,2]. Although COVID-19 is primarily characterized by its respiratory symptoms, it is now clear that the virus also affects the digestive system, with symptoms including diarrhea, nausea, vomiting, and diminished appetite [3,4]. About 50% of COVID-19 patients have detectable SARS-CoV-2 RNA in their feces [5,6], suggesting that the virus replicates in the gastrointestinal (GI) tract [7]. The evidence of viral shedding through feces is another strong indicator of GI involvement and raises the possibility of fecal–oral transmission [8]. Moreover, SARS-CoV-2 infection exerts gut tropism that is characterized by an acute inflammatory response that potentially deteriorates the course of human inflammatory bowel disease (IBD) [9]. Therefore, insights into the pathogenic mechanisms of COVID-19 in the GI tract will aid efforts to improve the prevention, diagnosis, and treatment for these patients.

The SARS-CoV-2 virion binds to the angiotensin (Ang)-converting enzyme 2 (ACE2) receptor via its spike (S) protein N-terminal S1 subunit, which is subsequently cleaved by the host cell androgen-induced transmembrane serine protease 2 (TMPRSS2). This process exposes the C-terminal S2 subunit of the S protein, inducing virus–cell fusion and viral spread in the infected host [10,11,12,13]. Other human coronaviruses, such as HCoV-229E, MERS-CoV, SARS-CoV, and the influenza virus also use TMPRSS2 to facilitate their cellular entry [14,15,16]. In addition, TMPRSS2 is abundantly expressed in the small intestine and in normal or carcinoid cells of the epithelial prostate and activates several substrates, including pro-hepatocyte growth factor/HGF, the protease-activated receptor-2/F2RL1, or matriptase/ST14, leading to disruption of the extracellular matrix and metastasis of prostate cancer cells [17]. While the respiratory tract is deemed the primary route of infection, *ACE2* expression in the GI tract is nearly 100-fold higher than in the respiratory tract [9]. Within the gut, patients with IBD display further elevated *ACE2* expression, which is more pronounced in Crohn’s disease compared with patients with ulcerative colitis [9,18,19]. However, it remains unknown whether patients with IBD or other inflammatory conditions are at increased risk of contracting SARS-CoV-2 due to immune dysregulation. Therefore, elucidating the underlying genetic regulatory mechanisms of *TMPRSS2* and *ACE2* co-expression will help to inform the prevention and treatment of GI disorders in COVID-19 patients. Furthermore, whether the synergistic action of other co-expressed genes in the GI tract also affects digestive dysfunction during COVID-19 remains to be explored.

The BXD family of strains carrying distinct mosaics of the B and D parental haplotypes, which were derived from C57BL/6J (B6) and DBA/2J (D2) strains, was constructed as a high-power resource for experimental precision health care [20]. The BXD family consists of approximately 152 BXD fully inbred strains that segregate ~6 million genetic variants and thus can be used as a replicable and extensible reference panel [21,22]. Each BXD strain is represented by a stable inbred strain (males and females are isogenic with the sole exception of the Y chromosome) that can be replicated in large numbers to reduce technical and environmental sources of variance. We successfully used the BXD family to perform the genetic dissection of *Ace2* expression variation in the heart and lung [23] and to expand our understanding on causal models of viral infection and signaling that is relevant to cardiovascular and respiratory diseases.

In this study, we took advantage of the exon array expression data on *Tmprss2* and *Ace2* in GI tissues from 50 members of the BXD family that are available through our GeneNetwork.org website [24,25]. Using these data and a systems genetics approach, we identified the *Tmprss2*- and *Ace2*-correlated genes, potential networks, and candidate genes that were up- or downstream regulators and that may contribute to the GI function that was associated with post-viral infections and related GI complications.

## 2. Materials and Methods

### 2.1. Animals and Tissue Collection

A total of 50 mouse strains comprising 46 BXDs, two parental B6 and D2 strains, and their two reciprocal F1 hybrid strains, were used in this study. Most strains that were used for the generation of GI transcriptome data contained one male and one female mouse. All mice were fed a chow diet throughout life after weaning until euthanasia at around 2–3 months of age. All the animals were housed in an individually ventilated cage (IVC) system in the Animal Care Facility at the University of Tennessee Health Science Center (UTHSC), Memphis, TN, USA. The vivarium was kept under a 12 h light/12 h dark cycle at a controlled room temperature of 20 ± 2 °C and humidity of 35%. The animals had *ad libitum* access to food and water throughout the experiment.

For the GI tissue collection, anesthetized animals were sacrificed using cervical dislocation. Approximately two equal-sized segments of the small intestine were pooled per animal: one was taken from the proximal jejunum and the other from the distal ileum. All animal procedures were carried out in accordance with the UTHSC guidelines on the humane treatment of experimental animals and with the explicit approval of the Institutional Animal Care and Use Committee (IACUC).

### 2.2. RNA Isolation and Transcriptome Data Generation

Following isolation, the GI tissues were shattered in liquid nitrogen and the total RNA was isolated using a miRNeasy Mini Kit (Qiagen, Hilden, Germany) according to the manufacturer’s instructions. Briefly, approximately 30 mg tissue was added to a 2 mL tube containing 700 µL of QIAzol lysis reagent and a 5 mm stainless steel bead (Qiagen, Hilden, Germany). The tissue was homogenized in Tissue Lyser II (Qiagen, Hilden, Germany) for 2 min at a rate of 30 r/s and incubated for 5 min. Then, 140 μL of chloroform was added to the homogenate, which was then shaken vigorously for 15 sec, followed by centrifugation at 12,000× *g* for 15 min at 4 °C. Subsequently, 280 µL of the supernatant was transferred to a new collection tube containing 500 µL of 100% ethanol. The mixture was loaded onto an RNeasy Mini Spin Column (Qiagen, Valencia, CA, USA) and purified once with Buffer RWT and twice with Buffer RPE. The concentration and purity of the RNA were determined using a NanoDrop spectrophotometer (Thermo Fisher Scientific, Wilmington, DE, United States). The RNA integrity (RIN) was assessed using an Agilent 2100 Bioanalyzer (Agilent, Santa Clara, CA, United States). Samples that passed the quality control (RIN > 8.0) were run on Affymetrix Mouse Gene 1.0 ST arrays at the UTHSC.

### 2.3. Data Preprocessing

Raw microarray data were first normalized using the Robust Multichip Array (RMA) method [26]; then, the data were logged and Z-normalized [27]. To remove negative values from the tables, we shifted the mean to 8 units and increased the standard deviation of each array data-set to two units such that a 2-fold difference in expression corresponded to an approximately 1 unit increase in expression as judged by the spike-in controls [28].

### 2.4. Expression Data and FAIR Data Access

Affymetrix MoGene 1.0 ST transcript expression data for the GI tract was uploaded to the GeneNetwork (GN) (genenetwork.org) in two forms: the first at the “gene” level, essentially a consensus of all exons that were probed, and the second at the exon level itself. All of the analyses in this paper used the gene-level consensus expression estimates.

Expression data for *TMPRSS2*/*ACE2* [29] and *Tmprss2*/*Ace2* [30] in various tissues of human and mouse were obtained from the National Center for Biotechnology Information website (https://www.ncbi.nlm.nih.gov/, accessed on 15 December 2020).

### 2.5. Expression Quantitative Trait Locus (eQTL) Mapping

The eQTL mapping of the transcripts was performed in the GN, as described in our previous work [23,31,32]. Two methods were used for the eQTL mapping. The first was the conventional interval mapping using a fast linear mapping method [33]; the second was the genome-wide efficient mixed-model association (GEMMA), which is a slower but more accurate method and incorporates a correction for differential kinship between strains [34]. The former yields a likelihood ratio statistic (LRS) score to measure the confidence of linkage between the observed phenotype and a genomic region, whereas GEMMA outputs a conventional −logP of linkage as in most genome-wide association studies.

### 2.6. Microbiome Analysis and Data Access

Cecal contents were collected from adult males of 32 BXD strains and parental strains (3–5 animals/strain) that were fed a standard chow diet, as previously described [35]. Briefly, isolated DNA underwent V5–V6 16S amplification, followed by next-generation sequencing on an Illumina MiSeq platform. Operational taxonomic units (OTUs) were assigned at 98% similarity in USEARCH v8.1.1861. Taxonomic assignment was performed in CLASSIFIER (rdp_classifer_v2.10.1) using Ribosomal Database Project (RDP). Sequence data are available at the NCBI Sequence Read Archive (SRA) under the BioProject number PRJNA557049.

### 2.7. Correlation Analysis

Genetic correlation analysis was performed with the Pearson correlation coefficient to identify gene–phenotype and gene–gene associations. For the gene–phenotype correlation analysis, we correlated phenotypes of operational taxonomic units (OTUs) that were stored in our GN database to the mRNA level of *Tmprss2* and *Ace2* in the GI tract. The resulting *p*-values < 0.05 were considered significant. Next, we identified the *Tmprss2* and *Ace2* genetically correlated genes across the GI transcriptomes, respectively. Genes that were significantly correlated with the expression of *Tmprss2* and *Ace2* (*p* < 0.05) were selected.

### 2.8. Weighted Gene Co-Expression Network Analysis (WGCNA)

WGCNA is a method to find co-expressed gene networks and explore the associations between genes and between gene networks and phenotypes of interest, as well as hub genes in the network. In this study, gene co-expression networks were constructed using the WGCNA package in R [36] according to the recommended tutorials (horvath.genetics.ucla.edu/html/CoexpressionNetwork/Rpackages/WGCNA/Tutorials/) in which both the *Ace2* and *Tmprss2*-correlated genes (Pearson correlation *p*-value < 0.05) were used as the input. The network construction mainly included the following four steps: (1) defining the gene expression correlation matrix, (2) selecting the soft threshold β and transforming the expression correlation matrix into an adjacency matrix, (3) converting the adjacent matrix to a topologically overlapping matrix and then to the dissimilarity matrix, and (4) hierarchical clustering of the dissimilarity matrix to obtain a clustering tree and refine with dynamic hybrid cutting.

### 2.9. Gene Set Enrichment Analysis

Gene set enrichment analysis was performed using WebGestalt (http://www.webgestalt.org, accessed on 15 December 2020) [37] to investigate the gene ontology (GO, biological processes), Kyoto Encyclopedia of Genes and Genomes (KEGG) pathway, and Mammalian Phenotype Ontology (MP) annotations. The *p*-value that was generated from the test was automatically adjusted to account for multiple comparisons using the Benjamini and Hochberg correction [38]. A minimum overlap of 5 genes and a false discovery rate (FDR) < 0.05 was required to determine the genes that were significantly overrepresented in those categories.

### 2.10. Protein–Protein Interactions (PPI) Analysis

To further discover the key co-expressed regulators in the WGCNA module, we constructed and evaluated the PPI network with NetworkAnalyst 3.0 (www.networkanalyst.ca, accessed on 15 December 2020) [39,40] using the International Molecular Exchange (IMEx) [41] Interactome database with default settings. The IMEx consortium is a publicly available database of curated and nonredundant sets of protein interactions.

## 3. Results

### 3.1. Tmprss2 and Ace2 mRNA Levels in Human and Mouse Tissues

**Figure 1:**
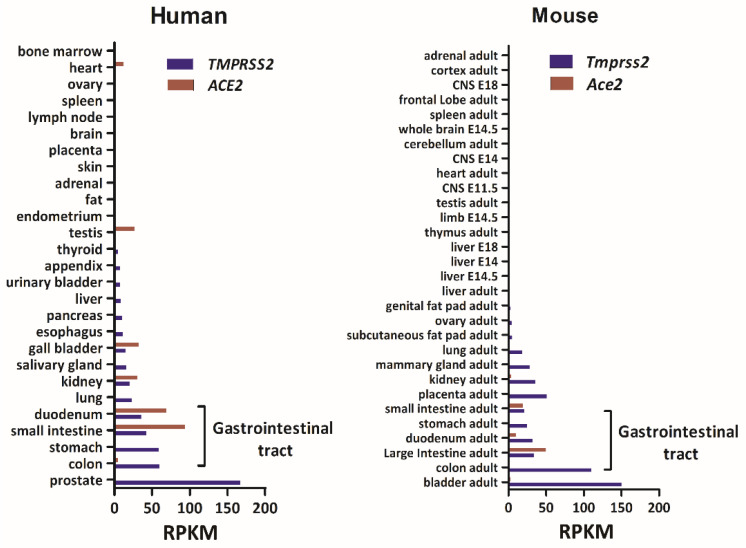
Bar charts of the mRNA levels of *TMPRSS2* and *ACE2* in human and mouse tissues. Data on mRNA levels of these two genes across the tissues were obtained from the National Center for Biotechnology Information website (https://www.ncbi.nlm.nih.gov/, accessed on 15 December 2020). The *x*-axis indicates the gene expression level in reads per kilobase per million mapped reads (RPKM) units. The *y*-axis indicates the tissues.

In this study, we first investigated the expression levels of *TMPRSS2* and *ACE2* in a variety of tissues for both human and mouse that were deposited at the National Center for Biotechnology Information website (https://www.ncbi.nlm.nih.gov/, accessed on 15 December 2020). The database consisted of human RNA-seq data representing 27 different tissues that were collected from 95 individuals [29] and a mouse RNA profiling dataset generated by the Mouse ENCODE project [30]. As shown in Figure 1, *TMPRSS2* was highly expressed in the prostate and GI tract, with an average expression of 49.25 reads per kilobase per million mapped reads (RPKM) in the GI tract compared to 12.77 RPKM in other tissues. The average mRNA level (RPKM) of *ACE2* was 42.15 in the GI and 4.86 in other tissues. The high expression levels of both of these genes in the mouse GI tract were similar to that in humans.

### 3.2. eQTL Mapping Identified a Common Regulating Locus for Tmprss2 and Ace2

**Figure 2:**
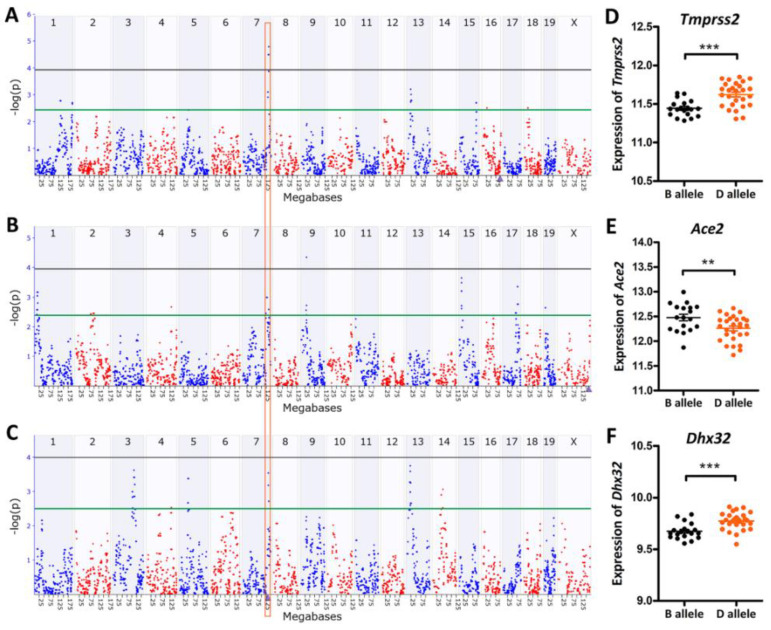
eQTL mapping of *Tmprss2*, *Ace2*, and *Dhx32* in the BXD strains. Manhattan plots of the genome-wide (**A**) *Tmprss2*-, (**B**) *Ace2*-, and (**C**) *Dhx32*-regulated genomic loci. eQTL mapping was performed with GEMMA on the GN. The *x*-axis denotes a position on the mouse genome in megabases (Mb). The *y*-axis indicates the −log(*p*) score, a measurement of the linkage between gene expression and genomic region. The purple triangle on the *x*-axis indicates the genomic position of the gene. The grey and green horizontal lines indicate the significant and suggestive threshold of the −log(*p*) scores for eQTL mapping, respectively, which were 3.95/2.41 for *Tmprss2*, 3.95/2.37 for *Ace2*, and 3.93/2.41 for *Dhx32.* The mRNA levels of (**D**) *Tmprss2*, (**E**) *Ace2*, and (**F**) *Dhx32* were significantly different between the B and D alleles at 133.997 Mb on Chr 7 (rs13479540) via an unpaired *t*-test. *** *p* < 0.0001 and ** *p* < 0.01. The gene expression values were log2-transformed.

**Figure 3:**
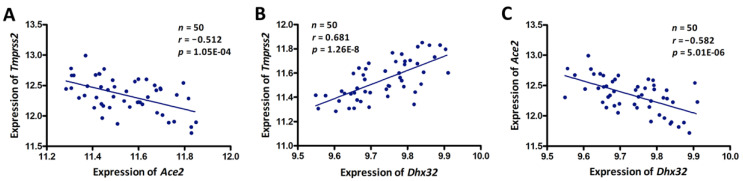
Scatter plots of the correlations between (**A**) *Tmprss2* and *Ace2*, (**B**) *Tmprss2* and *Dhx32*, and (**C**) *Ace2* and *Dhx32*. The Pearson correlation coefficient was used to determine the relationship. The Pearson correlation *r* and *p*-values are indicated in the figure. The gene expression values were log2-transformed.

The average mRNA level of *Tmprss2* in the GI tract across the BXD strains was 11.54 ± 0.16 SD. BXD50 and BXD62 mice had the lowest and highest expressions with 11.28 and 11.84, respectively. Similar to *Tmprss2*, *Ace2* with a mean expression of 12.34 ± 0.28 SD was also found to be highly expressed in the GI tissues of BXDs. A significant eQTL for *Tmprss2* was mapped to chromosome (Chr) 7 at 133.997 Mb (−log(*p*) = 4.77) using both GEMMA and fast linear mapping methods (Figure 2A). This locus was distantly located from the genomic location of *Tmprss2* (Chr 16 at 97.56 Mb), suggesting that it was a *trans*-acting eQTL. Furthermore, the corresponding eQTL mapping of *Ace2* in the GI tract also revealed a signal at the same location on Chr 7 (Figure 2B). The genomic location of *Ace2* (Chr X at 164.14 Mb) was also distant from the mapped location on Chr 7, indicating that it was a *trans*-acting eQTL for *Ace2*. Moreover, we found a significant negative correlation (*r* = −0.512, *p* = 1.05 × 10^−4^) between *Tmprss2* and *Ace2* expression in the GI tract of the BXD strains (Figure 3A). Therefore, it is reasonable to speculate that there is a common regulator candidate for these two genes at ~134 Mb on Chr 7.

### 3.3. Dhx32 was a Candidate Upstream Regulator for Tmprss2 and Ace2

The 1.5-LOD interval of Chr 7 eQTL encompassed a 2.5 Mb region from 132.26 to 134.74 Mb. We identified 14 genes (Table 1) in the QTL region. To further prioritize the candidate regulator, we performed eQTL mapping for these 14 genes. This resulted in the identification of *Dhx32* as the only *cis*-regulated gene with a –log(*p*) score of 3.53 on Chr 7 at 133.721 Mb (Figure 2C). This peak position was identical to that of *Tmprss2* and *Ace2* eQTL (Figure 2A,B).

Moreover, we investigated the expression correlation between the 14 candidate genes and *Tmprss2* and *Ace2* and we found that only *Ctbp2* and *Dhx32* showed a significant correlation with *Tmprss2* and *Ace2* (Table 1), respectively. By comparing the DNA sequence differences between the B6 and D2 mice, two genes (*Ctbp2* and *Adam12*) were identified to harbor missense variants. Next, we grouped the mice according to their genotype (B and D type) at the eQTL peak position (rs13479540, Chr 7 at 133.997 Mb). Statistical analysis revealed that the mRNA levels of *Dhx32* were significantly different (*p* < 0.01) between the B and D alleles (Figure 2F), as were the *Tmprss2* and *Ace2* levels (Figure 2D,E). Specifically, the BXD strains carrying the D allele expressed higher levels of *Tmprss2* and *Dhx32* while expressing lower levels of *Ace2*. Concomitantly, the expression of the *Dhx32* transcript showed a positive correlation with that of *Tmprss2* (*r* = 0.698, *p* = 3.42 × 10^−9^, Figure 3B), whereas it exhibited a negative correlation with *Ace2* expression (*r* = −0.560, *p* = 1.41 × 10^−5^, Figure 3C) in the BXD GI tissues. Taken together, we suggest that *Dhx32* is a candidate common upstream regulator of *Tmprss2* and *Ace2*.

Besides the eQTL on Chr 7, we also identified other signals on several chromosomes, including eQTLs located on Chr 13 for *Tmprss2*, and Chr 1, 9, 15, and 17 for *Ace2*. Of note, although the eQTL for *Ace2* on Chr 9 at 29.9 Mb achieved statistical significance with a -log(*p*) score of 4.33 (Figure 2B) identified by GEMMA, no significant or suggestive eQTL for *Ace2* was identified at this location via genetic mapping with a fast linear mapping method. Hence, there could be a false positive eQTL for *Ace2* at this location. In addition, by screening the genes in the 1.5-LOD region of this eQTL, we identified only two genes, namely, *Ntm* and *Snx19*. However, these two were neither *cis*-regulated nor correlated with the expression of *Ace2* in the GI tract (*p* > 0.05). Finally, no nonsynonymous variants were found in these two genes in the BXD mice. Therefore, the current evidence is insufficient to support *Ntm* or *Snx19* as upstream regulators for the expression of *Ace2*.

### 3.4. Genetic Correlations between Tmprss2 and Ace2 and GI Microbiota

**Figure 4:**
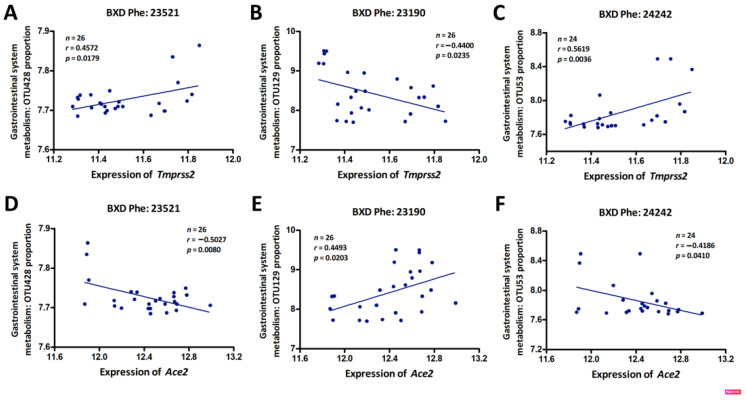
Scatter plots of the correlations between *Tmprss2* or *Ace2* expression and GI system microbiota phenotypes: OTU428 (**A**,**D**), OTU129 (**B**,**E**), and OTU53 (**C**,**F**). The Pearson correlation coefficient was used to determine the relationship. The Pearson correlation r and *p*-values are indicated in the figure. The gene expression levels were log2-transformed.

The GI system harbors a diverse microbiota that plays important roles in the host metabolism and the immune response to infection or inflammation [42]. While diet is the dominant driver of microbiota community dynamics, genetics can also shape host–microbial interactions and gut ecology [43,44]. We specifically hypothesized that variable expression in *Tmprss2* and *Ace2* in BXD animals would correlate with altered GI microbiota abundance in our animals that were fed a standard diet. To examine this, we sequenced the cecum microbiome of 32 BXD and their parental strains and generated operational taxonomic units (OTUs) at the 98% similarity cutoff. We identified three GI bacterial OTUs with significant gene correlations. Specifically, OTU428 (Firmicutes, Clostridiales, Ruminococcaceae, Ruminococcus) and OTU53 (Bacteroidetes, Bacteroidales, S24-7) (Figure 4A,C) were positively correlated with *Tmprss2* expression, while OTU129 (Firmicutes, Clostridiales) had a negative correlation with *Tmprss2* (Figure 4B). Moreover, these three microbial taxa were significantly correlated with the expression of Ace2, however, inversely when compared to the correlations with Tmprss2 expression (Figure 4D–F). These data suggest that the microbial abundance of these taxa may have been influenced directly or indirectly by the expression of *Tmprss2* and *Ace2* in the GI tract. These three microbiota features can be found on our GN website with the accession numbers 23521, 23190, and 242423, respectively.

### 3.5. Weighted Gene Co-Expression Network Analysis (WGCNA)

**Figure 5:**
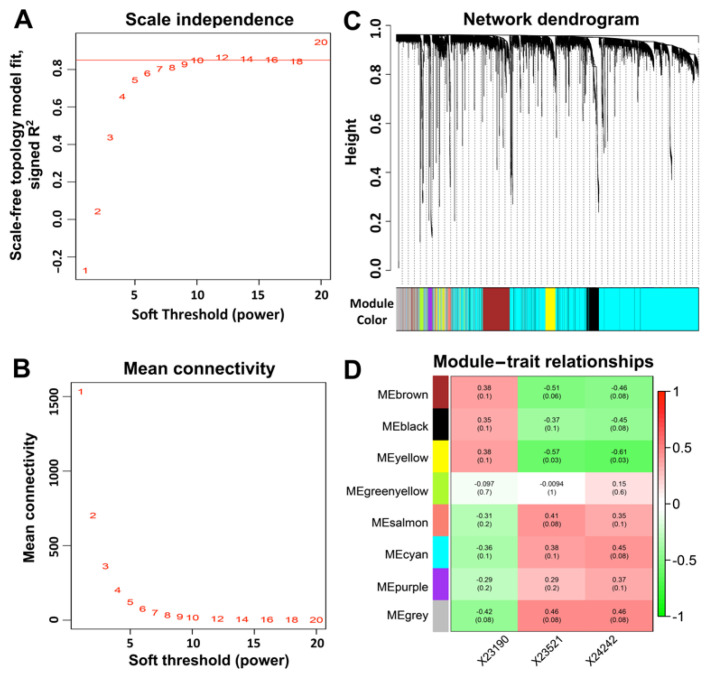
WGCNA modules that were associated with *Tmprss2* and *Ace2* expression. (**A**) Soft thresholding index R^2^ as a function of the soft-thresholding power β. A β = 12 indicated a scale-free topology. (**B**) Mean connectivity (degree) as a function of β. (**C**) Eight co-expression modules were identified from the ~4100 transcripts that co-varied with both *Tmprss2* and *Ace2* in the GI transcriptome data using dendrogram branch cutting. (**D**) Associations (Pearson correlation r with FDR in parentheses) between module eigengenes and GI microbiota phenotypes.

To understand the biological processes and gene pathways of *Tmprss2*- and *Ace2*-correlated genes, we next performed a gene–gene correlation analysis using the GN. In total, 15,017 and 7887 transcripts were correlated with *Tmprss2* and *Ace2*, respectively, among which, ~4100 transcripts co-varied with both *Tmprss2* and *Ace2*. Next, we constructed unsigned co-expression modules using WGCNA with a soft-thresholding power of 12 to achieve a scale-free distribution (Figure 5A,B). This parsed the ~4100 transcripts into eight modules (Figure 5C,D), including module M1 (brown, 438 transcripts), M2 (black, 267 transcripts), M3 (yellow, 217 transcripts), M4 (bright green, 51 transcripts), M5 (salmon, 41 transcripts), M6 (cyan, 2569 transcripts), M7 (purple, 60 transcripts), and M8 (grey, 488 transcripts). In addition, we summarized each module’s eigengenes (the first principal component of a given module) and correlated them against the three GI microbiota traits that were associated with both *Tmprss2* and *Ace2* (Figure 5D). We found that module M3 was significantly correlated (FDR < 0.05) with both OTU428 and OTU53. It is noteworthy that both *Tmprss2* and *Ace2* are located within this module.

**Figure 6:**
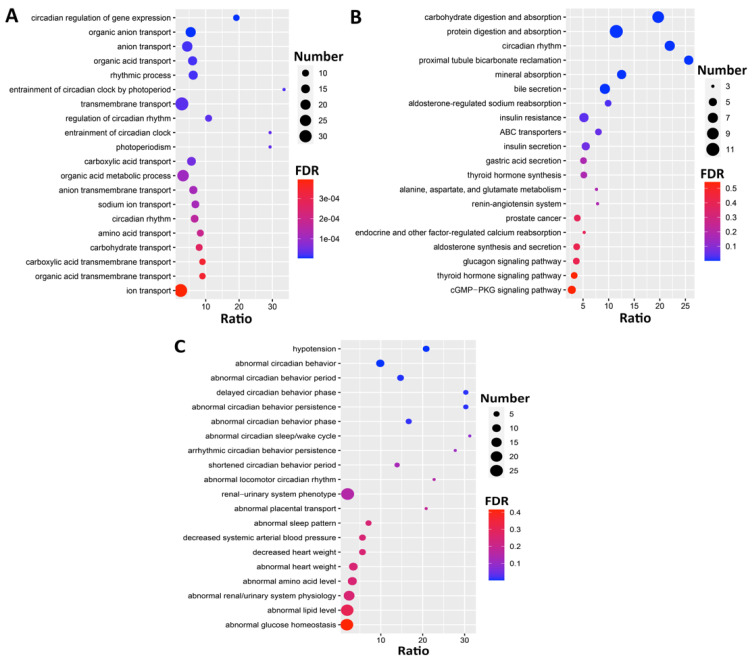
Bubble charts representing the enrichment results of top 20 (**A**) GO, (**B**) KEGG, and (**C**) Mammalian Phenotype Ontologies for the genes in the M3 module. The *x*-axis represents the enriched ratio and the *y*-axis represents enriched pathways/terms. The size of each dot represents the number of genes and the color indicates the *p*-value. The enriched ratio is defined as the number of observed genes divided by the number of expected genes from the annotation category in the gene list.

To investigate the biological functions associated with module M3, we performed GO, KEGG, and phenotype enrichment analyses. The GO enrichment (Figure 6A) showed that the M3 module genes were significantly enriched in circadian-rhythm-related terms, such as circadian regulation of gene expression (GO:0032922), rhythmic process (GO:0048511), and circadian rhythm (GO:0007623). Similarly, the KEGG enrichment results (Figure 6B) showed that the genes were not only associated with the circadian rhythm pathway (mmu04710) but were also significantly involved in carbohydrate digestion and absorption (mmu04973) and protein digestion and absorption (mmu04974). Additionally, the Mammalian Phenotype Ontologies enrichment (Figure 6C) indicated the significant association of these with circadian-behavior-related categories, such as abnormal circadian behavior (MP:0020467), abnormal circadian sleep/wake cycle (MP:0020478), and arrhythmic circadian behavior persistence (MP:0020472).

Further, the results demonstrated that *Tmprss2* was significantly involved in the “prostate cancer” KEGG pathway, as well as in the “import into cell” term with *Per2*, *Atp1a1*, and *Arrb1* (GO:0098657, FDR = 3.39 × 10^−5^). Similarly, *Ace2* was significantly involved in the well-known “renin–angiotensin system” pathway (mmu04614), the “protein digestion and absorption” (mmu04974, FDR = 3.5 × 10^−7^) pathway with *Atp1a1*, the “organic anion transport” term (GO:0015711, FDR = 7.33 × 10^−10^) with *Per2,* and the “hypotension” phenotype (MP:0001596, FDR = 2.99 × 10^−7^) with *Ppara* and *Arntl*.

### 3.6. Protein–Protein Interactions (PPI) Subnetwork

**Figure 7:**
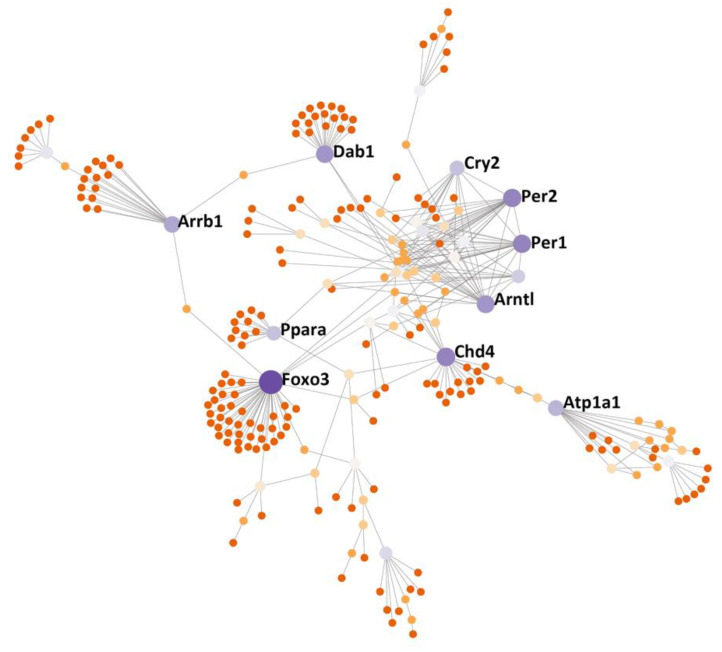
PPI subnetwork of the genes from the M3 module that was identified using WGCNA. The subnetwork was constructed and evaluated using NetworkAnalyst 3.0 (www.networkanalyst.ca, accessed on 15 December 2020) in which the International Molecular Exchange (IMEx) Interactome database was used. The nodes in the network represent genes and key node genes are indicated by gene symbols.

To further dissect the potential genetic PPI of the M3 module genes, we uploaded these genes into NetworkAnalyst 3.0 (https://www.networkanalyst.ca, accessed on 15 December 2020) and searched for the PPIs [39,40]. We identified a PPI subnetwork, which included several hub nodes (Foxo3, Chd4, Per1, Per2, Arntl, Dab1, Atp1a1, Arrb1, Cry2, and Ppara), as shown in Figure 7.

## 4. Discussion

Several studies showed that a significant proportion of patients with COVID-19 initially present with atypical symptoms that are indicative of GI involvement, such as vomiting, diarrhea, or abdominal pain during the early phases of the disease. Oftentimes, these precede respiratory symptoms [4,5,45]. In addition, a remarkably higher proportion of COVID-19 patients with GI symptoms (25%) progresses to having a more severe disease than the overall patient population (10.4%) [46]. Sun et al. investigated the prevalence and outcomes of acute gastrointestinal injury (AGI) in critically ill COVID-19 patients and the results showed that the AGI incidence was 86.7%. Furthermore, GI dysfunction imparts changes in intestinal microbes and an increase in inflammatory cytokines [47]. Patients with worse AGI grades had worse clinical severity variables and higher septic shock incidence and 28-day mortality [48], highlighting the significance of GI symptoms in predicting COVID-19 severity and outcome [49]. In this study, we aimed to study the genetic regulatory mechanisms of *Tmprss2* and *Ace2* that function as the gatekeepers for SARS-CoV-2 infection. We employed a systems genetics approach for investigating GI transcriptomes and phenotypes of BXD strains that are used as a GRP. Our results demonstrated that both *Tmprss2* and *Ace2* were highly expressed in GI tissues and significantly negatively correlated with each other. We explored their potential upstream regulatory genes using eQTL mapping and explored the gene co-expression networks.

### 4.1. Dhx32 Was the Upstream Regulator of Tmprss2 and Ace2

The present study identified *Dhx32* as a possible upstream regulatory gene for *Tmprss2* and *Ace2*. The TMPRSS2 protease competes with the metalloprotease ADAM17 for ACE2 processing, but cleavage of the S1 subunit of SARS-CoV-2 S protein by TMPRSS2 promotes S-mediated cellular entry [50]. Although additional molecular experimentation is needed for validation, a spatial relation between *Tmprss2* and *Ace2* may help to explain our finding that *Tmprss2* was negatively correlated with the *Ace2* expression in the GI tract. A previous study identified colorectal cancer (CRC) as unique amongst human malignancies owing to its co-expression of higher levels of both *ACE2* and *TMPRSS2* than in normal tissues [51]. Furthermore, data suggest that a proportion of healthy individuals are susceptible to SARS-CoV-2 intestinal infection and that patients with CRC may be at even greater risk of infection [52]. Of note, the overexpression of DHX32 contributes to the growth and metastasis of CRC [53,54] through the DHX32-induced upregulation of *VGFA* (vascular endothelial growth factor A) at the transcription level and stabilization of β-catenin [55]. In our study, the expression of *Dhx32* significantly correlated with the expression of *Tmprss2* and *Ace2* in the GI tract of BXD mice. Based on the eQTL mapping results, we propose that *Dhx32* may be a common upstream regulator for *Tmprss2* and *Ace2*, although other upstream regulators of *Tmprss2* or *Ace2* on other chromosomes cannot be ruled out.

### 4.2. GI Microbiota and COVID-19

Dysbiosis or decreased diversity in the gut microbiota during aging was postulated as the reason for older adults being at a higher risk for severe COVID-19 illness [56]. Hashimoto et al. reported that ACE2 links amino acid malnutrition to microbial ecology and deficiency in murine *Ace2* results in highly increased susceptibility to intestinal inflammation induced by epithelial damage [57]. In our study, the expression of *Ace2* and *Tmprss2* in GI tissues significantly correlated with key members of GI microbiota in BXD mice. Interestingly, other studies observed that *ACE2* and *TMPRSS2* gene expressions were associated with commensal microbiota in matched normal tissues, particularly from CRCs, with distinct bacterial signatures showing strong associations [58]. A meta-analysis also revealed that lung cancer and colorectal cancer patients are more susceptible to SARS-CoV-2 infection [59]. A previous study showed that the gut microbial profile of the BXD strains is dominated by *Firmicutes* and *Bacteroidetes* and displays substantial variability across strains [35]. Recent metagenomic sequencing of the BXD family cecum microbiota revealed important differences in bacterial composition across strains and revealed that diet modulates cecum bacterial diversity and physiological phenotypes across the GRP [60]. Thus, the association between the host genetic background and the microbiota community composition and diversity in the GI tract are well supported. Further, environmental conditions, such as diet, also shape the composition of the GI microbiota, which together are likely to shape individual susceptibility differences and outcome complexity in COVID-19 patients.

### 4.3. Circadian Rhythms Involved in GI Function and Contribution to COVID-19

All gene set enrichment analyses (GO, KEGG, and MP) demonstrated a significant association of the genes in the M3 module that was identified using WGCNA with the circadian-rhythm-related terms. Similarly, 7 proteins (Foxo3, Per1, Per2, Arntl, Dab1, Cry2, and Ppara) in the PPI subnetwork out of 10 hub genes of the M3 module were associated with circadian-rhythm-related pathways or behavior. This suggested that the M3 module gene network may have been primarily involved in the regulation of circadian rhythms in the gut, and *Tmprrs2* and *Ace2* may have interacted with these circadian genes. As previously established, ACE2 catalyzes the conversion of AngII to Ang1–7, which acts as a vasodilator [61] and plays a critical role in the control of cardiovascular and renal functions by maintaining the physiological homeostasis of blood pressure and electrolyte balance [62]. AngII infusion was reported to influence clock gene expression and diminish diurnal rhythms of Ace/Ace2 mRNA ratios in the aorta, indicating a modulatory effect of AngII on tissue and the renin–angiotensin system [63]. Moreover, circadian rhythms regulate many gastrointestinal physiological processes, including cell proliferation, motility, digestion, absorption, mucosal secretions, and electrolyte balance [64]. Circadian rhythm was recently suggested as a regulator of viral infections, and in particular, experimental evidence supports the involvement of circadian rhythms in COVID-19 progression [65]. Supporting our results, circadian rhythm as an evolutionarily conserved pathway was suggested as a new target for reducing the risk of COVID-19 infection, as well as for developing diagnostic and therapeutic strategies [66,67].

## 5. Conclusions

In this study, we performed the genetic dissection of *Tmprrs2* and *Ace2* involved in SARS-Cov-2 virus–cell fusion and viral spread in the host and identified their up- and downstream regulators, mechanisms, pathways, and networks that may underlie the GI symptoms and complications in COVID-19. The results of our study suggest the roles of *Tmprss2* and *Ace2*, as well as other hub genes that were identified within the co-expression network in GI function and circadian rhythm regulation. These could represent important nodes that regulate infection risk, disease severity, and outcome in COVID-19 and warrant further investigation.

## Figures and Tables

**Table 1:** Lists of all upstream candidate genes in the Chr 7 QTL interval.

Gene ID	Gene Symbol	Location (Chr, Mb)	Mean Expression	Max LRS	Cis-eQTL	*Tmprss2* -*r*	*Ace2* -*r*	Nonsynonymous Variants
18242	*Oat*	Chr7: 132.558	12.3394	13.2	×	0.118	−0.186	×
20231	*Nkx1–2*	Chr7: 132.596	8.0721	13.5	×	−0.426	0.061	×
76429	*Lhpp*	Chr7: 132.611	9.4769	12.9	×	−0.35	−0.156	×
77938	*Fam53b*	Chr7: 132.712	9.5488	12.4	×	0.085	0.216	×
360216	*Zranb1*	Chr7: 132.950	8.2709	12	×	0.273	0.048	×
13017	*Ctbp2*	Chr7: 132.988	9.9881	12.2	×	0.693	−0.412	√
73808	*Tex36*	Chr7: 133.587	5.5396	8.9	×	−0.105	−0.049	×
214766	*Mmp21*	Chr7: 133.674	6.8655	11.7	×	−0.319	−0.009	×
22276	*Uros*	Chr7: 133.686	8.2217	9.9	×	0.468	−0.345	×
66165	*Bccip*	Chr7: 133.709	8.0386	13.4	×	0.636	−0.167	×
**101437**	***Dhx32***	**Chr7: 133.721**	**9.7337**	**15**	**√**	**0.681**	**−0.582**	**×**
66930	*Fank1*	Chr7: 133.777	7.2585	14.2	×	−0.176	−0.278	×
11489	*Adam12*	Chr7: 133.883	7.5060	16.9	×	−0.246	−0.144	√
330662	*Dock1*	Chr7: 134.671	9.5446	12	×	−0.521	0.325	×

## Data Availability

The gene expression data “UTHSC Mouse BXD Gastrointestinal Affy MoGene 1.0 ST Gene Level (Apr14) RMA” were generated in our lab and can be accessed at our GeneNetwork (GN) website (http://gn1.genenetwork.org/webqtl/main.py?FormID=sharinginfo&GN_AccessionId=539, accessed on 15 December 2020).
